# Medical Students’ Perspectives on Online Proctoring During Remote Digital Progress Test

**DOI:** 10.1007/s40670-021-01420-w

**Published:** 2021-09-30

**Authors:** Fleur L. Meulmeester, Eline A. Dubois, C. (Tineke) Krommenhoek-van Es, Peter G. M. de Jong, Alexandra M. J. Langers

**Affiliations:** 1 Center for Education in Medical Education, Leiden University Medical Center, Leiden, The Netherlands; 2 Department of Gastroenterology and Hepatology, Leiden University Medical Center, Leiden, The Netherlands

**Keywords:** Students’ perceptions, Proctoring, Online assessment, Examination, E-proctoring

## Abstract

Remote teaching and assessment are essential for current education. During online examination, online proctoring is often used as a surveillance tool. Little is known about student perceptions on online proctoring. Using an online questionnaire, we found that medical students worry most about unjustified invalidation of their exam due to unstable internet connection, background noise or webcam issues, and privacy issues. It is important to be aware of these worries as they may influence test results.

## Background

Online teaching and assessment have taken a prominent place in medical education since the coronavirus disease 2019 (COVID-19) pandemic. Given the pace at which educational institutes had to revise their strategies to ensure the continuation of their students' education, online teaching and assessment have developed considerably over the past year [1, 2]. In high stake online examinations, many universities apply proctoring [1–3]. Online proctoring includes multiple assessment tools that track the students’ behaviour during the exam [4]. Different proctoring systems, with different features, are available. For example, the Proctorio® system requires that the (single) webcam and microphone at the students’ workstation remain activated to check if the student takes the exam alone. Furthermore, the proctoring platform monitors the students’ device display via screensharing and screen recordings. The software also registers which programs and/or websites are opened during the exam and can highlight suspicious or inappropriate behaviour by placing a red flag. After finishing the recordings, the footage is checked by reviewers, who can inspect suspicious behaviour and report this to the exam committee.

Online proctoring is believed to be a good solution for fraud prevention during summative remote testing and often found essential for the continuation of students’ education and graduation. It is unclear how medical students experience this supervision. Yet, their perceptions could be of great importance for the further development of proctored assessment. The aim of the present study is to gain more insight in the medical students’ perspectives on online proctored examinations, in the setting of a progress test.

## Activity

At Leiden University, large-scale digital testing was introduced in 2019. A dedicated exam location with workstations in a secure network is now available for all university programs. The Faculty of Medicine at Leiden University Medical Center (LUMC) started using these facilities in September 2019. Due to the pandemic, online examinations at the exam location have been replaced by online examinations from home. The Proctorio® software was introduced as proctoring tool. Medical students took their first online proctored exam with Proctorio® in June 2020.

To assess medical students’ perspectives on online proctoring, we conducted an anonymous online survey using Qualtrics XM software (Qualtrics, Provo, UT) in September 2020. All students in either the bachelor or master phase of the medical curriculum at LUMC were invited to fill out the questionnaire. The survey was offered to the medical students directly after they finished an online, formative (non-proctored) national progress test from home. In the Netherlands, progress tests are administered in Dutch four times per year for all medical school students at all participating universities (at LUMC about 2000 students). All students receive exactly the same test while the pass/fail threshold increases stepwise from the first until the final year of the study program [5–7]. Under normal on campus circumstances, the progress tests are written, 4-h summative paper tests. Due to the initial lack of capacity to use online proctoring for this high number of students, the September 2020 progress test was administered as a 4-h formative test.

Before opening the questionnaire, the students were instructed to answer the questions imagining the progress test they just finished had been proctored. Participation was voluntary and did not affect the outcome of their test or credits; informed consent was not obtained as the questionnaire was primarily intended for quality assurance in anticipation of future online proctored progress tests. The questionnaire was offered after completion of the progress test, and there was no time limit for completing the questionnaire. Survey items are listed in Table 1. The majority of participating students had already experienced at least one digitally proctored exam with the software of Proctorio®. Descriptive statistics were generally used; to compare the answers between the different student cohorts, Pearson’s chi square test was used.

**Table 1:** Online proctoring experience survey items

Question or statement	Answer options
**1.** I am confident that my online progress test will not be invalidated	Totally agree/agree/neutral/disagree/totally disagree
**2.** I trust the way in which my footage will be checked for cheating during an online progress test	Totally agree/agree/neutral/disagree/totally disagree
**3.** I am afraid my online progress test will be invalidated, even though I did not cheat	Totally agree/agree/neutral/disagree/totally disagree
**4.** During your progress online test, what could the online proctoring program consider to be cheating?*	Free text entry
**5.** In my opinion, universities should be allowed to use proctoring during the online progress test	Totally agree/agree/neutral/disagree/totally disagree
**6.** What is the reason you disagree with the use of proctoring during the online progress test?*	Free text entry
**7.** In my opinion, universities should be allowed to use proctoring during the online progress test if this prevents study delay	Totally agree/agree/neutral/disagree/totally disagree

*Questions were asked only when the student answered *disagree* or *totally disagree* to the previous statement. As the online progress test was offered in Dutch, all questions of the survey were also provided to the students in Dutch and afterwards translated for research purposes

## Results

### Test Anxiety and Issues

**Fig. 1:**
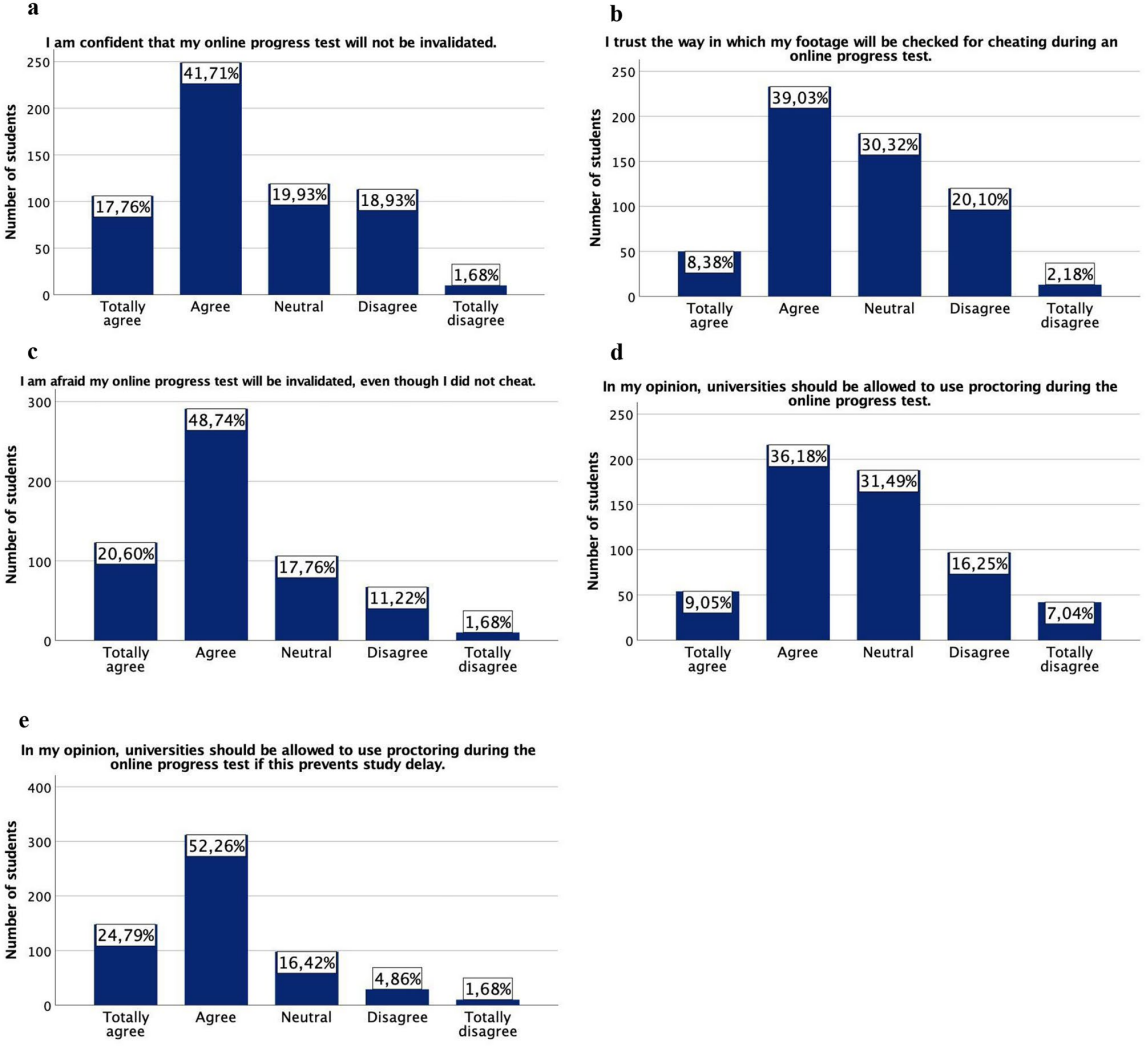
Distribution of student perceptions on online proctoring, stratified per question of the questionnaire (**a**–**e**). The bar charts represent the number of students that answered ‘totally agree’, ‘agree’, ‘neutral’, ‘disagree’, or ‘totally disagree’ to the questions of the online questionnaire. The total number of respondents is 597 (100%)

The survey was completed by 597 medical students (35.0% response rate). More than half of the respondents was confident that proctoring software would not invalidate their future online progress tests (*totally agree* or *agree*; 59.5%) (Fig. 1a). Furthermore, almost half of the students responded they trusted the way their footage will be checked for cheating (*totally agree* or *agree*; 47.4%) (Fig. 1b). Yet, almost 70% of all students were afraid that their progress test would be invalidated, even though they did not cheat during their online assessment (*totally agree* or *agree*; 69.3%) (Fig. 1c). Free text comments indicate that students are particularly worried about the possible impact of looking away from the webcam (371 students; 62.1%) and ambient noise from roommates, family, or neighbours (325 students; 54.4%). Other concerns are unstable internet connection (235 students; 39.4%) or a dysfunctional webcam (136 students; 22.8%). Overall, bachelor students were more confident that their test would not be invalidated compared to master students (*P* < 0.001; data not shown).

### Preferences

The majority of the respondents either approved of or were neutral towards the use of proctoring in online assessment of the progress test (*totally agree* or *agree*, 45.2%; *neutral*, 31.5%) (Fig. 1d). In the free text comments, 68 students mentioned violation of their privacy by proctoring as an argument for disapproval of proctoring applications. Furthermore, 31 students did not believe online proctoring to be trustworthy and would like to work with an alternative method for online progress testing. Again, internet malfunction and the anxiety that the proctoring software will incorrectly flag their behaviour — for example by looking away from the webcam — were the most frequently reported causes for disapproval. When the students were asked if they would approve the use of online proctoring when this would guarantee no study delay, the vast majority of students (*totally agree* or *agree*; 77.1%) approved and only 39 students (*disagree* or *totally disagree*; 6.5%) did not (Fig. 1e). Generally, more bachelor student approved the use of proctoring compared to master students (*P* < 0.001; data not shown).

## Discussion

Online teaching and assessment have taken a leap forward since the beginning of the COVID-19 pandemic. To enable the continuation of students’ education, universities had to implement online, remote exams, often accompanied by online proctoring. Multiple studies have shown that in non-proctored online exams, students seek assistance from friends or books, which underlines the need for some form of supervision during online summative tests [2, 8–10]. Previous studies have focused on test results and test anxiety in online assessment [3, 4, 11]. In an unproctored remote examination setting [11], students’ opinions were mixed: they either felt more at ease at home or reported higher test anxiety due to worries about unstable internet connection. Studies regarding students’ perceptions of on campus versus online, remote exams reported more stress amongst students taking the exam remotely [12, 13]. Another study, conducted in the pre-COVID era, found no overall difference in test results when comparing online proctored exams with “physically” surveillance exams, although some students with high trait test anxiety performed worse in proctored online testing [3]. Information about how students perceive online proctoring is lacking, as this has never been investigated in a large cohort.

Generally, we observed that medical students do not have a negative attitude towards online proctoring. Yet, two aspects should be considered. Firstly, whereas most students have confidence in a valid test result, even more students reported that they do worry about unjustified invalidation of their exam. This apparent discrepancy may be explained by the fact that students, albeit trusting the proctoring software in general, still experience an increased anxiety of being ‘accused’ of cheating during their online exam. The second aspect concerns violation of privacy. Interestingly, we found a high number of students reporting privacy issues, even though there were no specific questions about this topic in the questionnaire. The fear of unjustified invalidation of the exam and privacy issues may be partially solved by improved communication about what happens to the footage and the involvement of human judgement in the interpretation of the data generated by the proctoring software. Most students indicated that in the end, they would accept online proctoring if it prevents any study delay.

One of the strengths of the study is the relatively large number of respondents from both the bachelor and master medical curriculum. Furthermore, as the relatively short survey was sent out directly after a formative, non-proctored progress test, we hypothesized that the influence of test anxiety and post-test stress on the survey answers was minimized, whereas the limited length of the questionnaire maximized the response rate. A limitation of this study is the limited number of questions in the questionnaire, with only two possibilities to enter free text. Furthermore, we cannot rule out that the fact that the preceding test was a non-proctored, formative test influenced the answers of the students. Lastly, the questions focused particularly on cheating and anxiety, and therefore, other issues may be underexposed.

## Conclusions

In conclusion, our data suggest that most students are willing to accept online proctoring, especially if this may prevent study delay. However, a substantial percentage of the students reported fear of being wrongly accused of fraudulent behaviour (possibly leading to invalidation of the test result) and invasion of privacy. Improved communication and demonstrations to students about the handling of the footage and judgement of the recorded material may help to alleviate these worries. Since online education and (proctored) assessment will probably continue to be a part of the educational spectrum, even after the COVID-19 pandemic, it is essential to be aware of the students’ perceptions of online proctoring. Further research could explore these perceptions in more detail, particularly in proctored course examinations.
